# Supplementary material for: Transcriptomic Insights into Mechanisms of Early Seed Maturation in the Garden Pea (Pisum sativum L.)
Source: Cells. 2020 Mar 23;9(3):779. doi: 10.3390/cells9030779 (PMC7140803; doi:10.3390/cells9030779)

Sprint−2,20(2)

Global Summary

%DE = 0.26

genes with fdr < 0.2 = 540 (247 + / 293 −)

genes with fdr < 0.1 = 501 (235 + / 266 −)

genes with fdr < 0.05 = 459 (218 + / 241 −)

genes with fdr < 0.01 = 416 (199 + / 217 −)

genes in genesets = 0

<FC> = 0

<t−score> = 0

<p−value> = 0

<fdr> = 0.74

Portrait

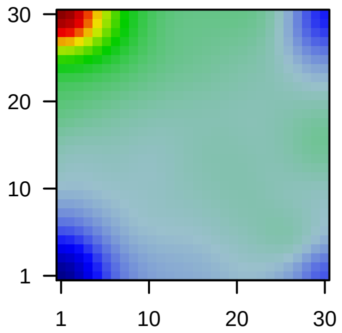

Top 100 DE genes

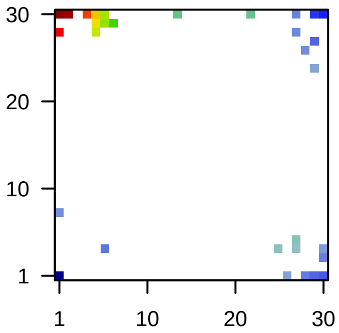

Global Genelist

Rank	ID	log(FC)	p−value	fdr	Metagene	Description
1	TRINITY_DN1	0.41	2e−16	2e−15	30 x 1	
2	TRINITY_DN2	−0.58	2e−16	2e−15	1 x 1	
3	TRINITY_DN9	−0.71	2e−16	2e−15	30 x 30	
4	TRINITY_DN7	−1.76	2e−16	2e−15	30 x 30	
5	TRINITY_DN5	−1.63	2e−16	2e−15	30 x 30	
6	TRINITY_DN5	−0.54	2e−16	2e−15	1 x 1	
7	TRINITY_DN5	1.11	2e−16	2e−15	1 x 30	
8	TRINITY_DN7	−1.24	2e−16	2e−15	1 x 1	
9	TRINITY_DN9	0.39	2e−16	2e−15	25 x 4	
10	TRINITY_DN4	0.54	2e−16	2e−15	1 x 28	
11	TRINITY_DN4	−0.84	2e−16	2e−15	1 x 1	
12	TRINITY_DN9	1.51	2e−16	2e−15	4 x 30	
13	TRINITY_DN9	−0.84	2e−16	2e−15	30 x 1	
14	TRINITY_DN9	−0.35	2e−16	2e−15	29 x 24	
15	TRINITY_DN9	0.44	2e−16	2e−15	4 x 30	
16	TRINITY_DN9	−0.47	2e−16	2e−15	30 x 30	
17	TRINITY_DN9	−0.86	2e−16	2e−15	30 x 30	
18	TRINITY_DN9	0.37	2e−16	2e−15	22 x 30	
19	TRINITY_DN9	0.88	2e−16	2e−15	27 x 4	
20	TRINITY_DN9	0.45	2e−16	2e−15	6 x 4	

p−values

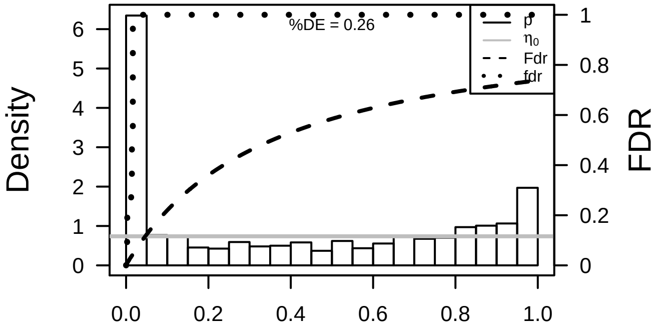

Supplement: Supplementary file 1 [file cells-09-00779-s001.zip › Supplementary materials/FileS4.zip!/FileS3/TFs - Results/Summary Sheets - Samples/Sprint.2.20.2..pdf]
